# Reorganization of Synaptic Connections and Perineuronal Nets in the Deep Cerebellar Nuclei of *Purkinje Cell Degeneration* Mutant Mice

**DOI:** 10.1155/2016/2828536

**Published:** 2015-12-27

**Authors:** M. Blosa, C. Bursch, S. Weigel, M. Holzer, C. Jäger, C. Janke, R. T. Matthews, T. Arendt, M. Morawski

**Affiliations:** ^1^Paul Flechsig Institute of Brain Research, University of Leipzig, Liebigstraße 19, 04103 Leipzig, Germany; ^2^Institut Curie, Centre de Recherche, CNRS UMR3348, Centre Universitaire, 91405 Orsay, France; ^3^Department of Neuroscience and Physiology, Sunny Upstate Medical University, 750 East Adams Street, Syracuse, NY 13210, USA

## Abstract

The perineuronal net (PN) is a subtype of extracellular matrix appearing as a net-like structure around distinct neurons throughout the whole CNS. PNs surround the soma, proximal dendrites, and the axonal initial segment embedding synaptic terminals on the neuronal surface. Different functions of the PNs are suggested which include support of synaptic stabilization, inhibition of axonal sprouting, and control of neuronal plasticity. A number of studies provide evidence that removing PNs or PN-components results in renewed neurite growth and synaptogenesis. In a mouse model for Purkinje cell degeneration, we examined the effect of deafferentation on synaptic remodeling and modulation of PNs in the deep cerebellar nuclei. We found reduced GABAergic, enhanced glutamatergic innervations at PN-associated neurons, and altered expression of the PN-components brevican and hapln4. These data refer to a direct interaction between ECM and synapses. The altered brevican expression induced by activated astrocytes could be required for an adequate regeneration by promoting neurite growth and synaptogenesis.

## 1. Introduction

The function of the nervous system is based on a precise composition and maintenance of a neuronal and synaptic network. The connectivity of the brain is formed during a period of enhanced plasticity in development when appropriate synaptic connections are stabilized in an activity dependent manner. In contrast, once the adult connectivity is established, plasticity of some synaptic contacts is greatly diminished. Functional alterations as they occur in many brain disorders are also accompanied by remodeling of neuronal structures, changes in neuronal activity, and loss of neuronal molecules [1–3]. A number of studies demonstrated that several extrinsic [4–7] and intrinsic [1–3, 8, 9] changes are associated with alterations in synaptic density or shape, dendritic outgrowth, and even extracellular matrix molecules. Especially a specialized form of the extracellular matrix, the perineuronal net, often shows alterations in neurodegenerative diseases [8–11] and acute brain injuries [7, 11–15] and is suggested to prevent regeneration. These perineuronal nets (PNs) enclose the cell bodies and the proximal dendrites of specialized neurons thereby embedding the contacting synaptic boutons [16–18]. PNs are composed of aggregating chondroitin sulphated proteoglycans (CSPGs), hyaluronan, hyaluronan binding link proteins (hapln), and tenascin-R [19–22]. CSPGs of PNs belong to the lectican family including the main members aggrecan, brevican, and neurocan, while aggrecan is prominently detected in PNs [23, 24]. Most of the PN-components are produced by neurons and glial cells, but a few constituents are made by only one of these cell types [25, 26]. PNs are involved in organizing extracellular space, modulating synaptic plasticity, and providing a special extracellular ionic milieu and synaptic stabilization [16, 27–32]. The formation and maintenance of PNs in a number of systems are activity dependent [31, 33–36]; thus they mainly occur at highly active neurons and altered activity disrupts PN formation [27, 34, 35, 37–42]. To analyze the potential role of PNs in degeneration/regeneration of slow denervation processes and to analyze the declining influence of synaptic input on PNs we use a mouse model for Purkinje cell degeneration (*pcd, pcd-3j/J model*). The pathology is caused by a mutation of the Nna1 gene [43, 44] encoding a protein also known as cytosolic carboxypeptidase 1 (CCP1) [45, 46], which has been demonstrated to be involved in the enzymatic deglutamylation of proteins, and in particular of tubulin [47]. It was therefore suggested that neurodegeneration in the* pcd* mouse is induced by a hyperglutamylation of microtubules in the affected neurons. In a rescue experiment the depletion of the tubulin tyrosine ligase-like protein 1 (TTLL1) [48] could partially prevent degeneration of the Purkinje cells (PCs) [47].

The PCs as part of the cerebellum are involved in motor coordination and posture control; as consequence in the pcd-3j/J model a loss of PCs leads to a moderate ataxia beginning at 3-4 weeks of age [43]. In addition, the degeneration of PCs is accompanied by the loss of cerebellar granule neurons [43, 49], olfactory mitral cells [50], some thalamic neurons [43], and alterations in retinal photoreceptors [50, 51]. Before PCs degenerate, which starts ~P18 and proceeds until ~P45, the PCs and their synaptic contacts show a normal development [52].

The GABAergic PCs receive virtually all input from within the cerebellum and provide the exclusive output of the cerebellar cortex, mainly inhibiting neurons of the deep cerebellar nuclei (DCN). The cells of DCN are a heterogeneous population of inhibitory and excitatory neurons [53–57], but only the large excitatory DCN neurons are surrounded by the condensed specialized extracellular matrix of PNs [25, 58–60]. However, it was repeatedly demonstrated that PN-associated neurons are protected against different neurotoxic insults and degenerative processes while neurons without a PN are not [8, 13, 61, 62].

Here we are investigating the integrity and expression of PNs and their components as well as the synaptic innervation and remodeling of DCN neurons after the degeneration of their main GABAergic input, the PC axons. The PN-associated DCN neurons showed an imbalance of inhibitory and excitatory innervations. We found a reduced GABAergic synaptic input and simultaneously these neurons receive an increased glutamatergic input. Further, the cytochemical analyses showed that the molecular composition of PNs has changed and revealed that brevican and hapln4 are prone to the degeneration processes and may influence the regeneration of the injured tissue.

## 2. Experimental Procedures

### 2.1. Animals

Data were collected from 11 pcd3j (C57BL/6-Agtpbp1^pcd-3j^/J, Stock # 003237) knock out mice and 11 wild type (*wt*) littermates (6 mice of each genotype for immunocytochemistry and 5 mice of each genotype for biochemistry) of both types of sex at the age of 4 months. Animals were genotyped as juveniles by PCR as described on* The Jackson Laboratory's* website (Genotyping protocol database of the Jackson Laboratory). They had free access to food and water and were maintained on a 12/12 h light-dark cycle under conditions of constant temperature (22°C). All animals used in this study were treated in agreement with the German law on the use of laboratory animals. The ethical guidelines of the laboratory animal care and use committee at the University of Leipzig were followed.

### 2.2. Cytochemistry

The animals were deeply anesthetized with CO_2_ and perfused transcardially with 10 mL 0.9% NaCl following 100 mL fixative containing 4% paraformaldehyde and 0.1% glutaraldehyde in 0.1 M phosphate buffer (pH 7.4). Brains were removed and postfixed in the same fixation solution overnight. The tissue was cryoprotected in 30% sucrose with 0.1% sodium azide, cut in 30 *μ*m thick slices with a cryomicrotome in frontal planes, and collected in phosphate buffer containing 0.1% azide.

Before staining, tissue was pretreated with 60% methanol containing 2% H_2_O_2_ for 20 min followed by a blocking step with a blocking solution containing 2% BSA, 0.3% milk powder, and 0.5% donkey serum in phosphate buffer for 1 h. All the antibodies (see Table 1) were incubated in blocking solution overnight at 4°C. The visualization was performed by standard fluorescent secondary antibodies (see Table 2). Identification of the investigated brain areas was made by brain atlas of mouse [72].

### 2.3. Imaging Procedures

Tissue sections were examined with a Zeiss Axiovert 200 M microscope (Zeiss, Jena, Germany) and a Zeiss confocal laser scanning microscope (Zeiss, Jena, Germany; LSM 510 meta). Confocal images of carbocyanine dye 2 (Cy2) fluorescence were obtained with the Argon laser (488 nm) and emission filter BP 505–530. The HeNe 1 laser (543 nm) and the emission filter BP 560–615 were used to detect the carbocyanine dye 3 (Cy3) fluorescence, respectively. Photoshop CS2 (Adobe Systems, Mountain View, CA, USA) was used to process the images with minimal alterations to the contrast and background.

### 2.4. Quantification

To estimate molecular alterations in* pcd* mice frontal sections were investigated and PN-bearing neurons of* nucleus interpositus* and* nucleus dentatus* of the DCN and the* nucleus vestibularis lateralis* were analyzed. The sections were labeled with anti-human aggrecan antibody (HAG7D4), the most comprehensive marker for PNs and additionally with GAD65/67 or vGlut1 and 2 for double immunofluorescence counting. The tissue was analyzed with a Zeiss Axiovert 200 M microscope equipped with a motorized stage (Märzhäuser, Germany) with MosaiX software and by means of a CCD camera (Zeiss MRC) connected to an Axiovision 4.6 image analysis system (Zeiss, Germany).

Counts were performed using the optical fractionator method [19, 37] on a Zeiss Axioskop 2 plus microscope (Jena, Germany) equipped with a motorized stage (Märzhäuser, Wetzlar, Germany), a Ludl MAC 5000 controller (LEP, Hawthorne, NY, USA), and a digital camera CX9000 (MicroBrightField, Williston, VT, USA). Stereo Investigator software 6 (MicroBrightField, Williston, VT, USA) was used to analyze the 30 *μ*m thick sections.

The contours of the DCN were outlined in the Stereo Investigator program using a 10x lens, cell diameter determination and synapse counts were performed using an oil-immersion 63x lens (1.4 numerical aperture).

Somatic boutons were counted from the cell surface of the large glutamatergic PN-bearing projection neurons (≥10 *μ*m diameter) up to a distance of 3 *μ*m in the periphery. For quantification of the boutons in the periphery, the cells were outlined at a distance of twofold diameter of the cell from the cell surface. Peripheral boutons were counted from the end of the somatic zone (≥3 *μ*m) to the outlined area.

### 2.5. SDS-PAGE and Western Blot Analyses

Mice were deeply anesthetized with CO_2_, decapitated, brains rapidly removed, and immediately frozen in liquid nitrogen. On a dry-ice cooled work plate the brains were cut in 2 mm frontal sections and DCN as precise as possible separated and stored in 2 mL tubes at −80°C until further proceeding. The DCN containing tissues of 5 mutant and 5* wt* mice were homogenized using an Ultra-Turrax tube drive (IKA), in homogenization buffer (20 mM Tris-HCl, 2 mM EDTA, 0.15 M NaCl, 5 mM NaF, 1 mM Na_3_VO_4_, and 2 mM MgCl_2_, pH 7.4) containing a protease inhibitor (Complete, Roche, Mannheim, Germany). The homogenate was centrifuged at 10.000 ×g for 14 min at 4°C, followed by determination of the protein concentration in the supernatant by using the BCA Assay. For discontinuous SDS-Page the supernatant containing 35 *μ*g proteins was mixed with 1x SDS sample buffer and denaturized at 70°C for 15 min. The proteins were separated on a 10% polyacrylamide gel and transferred to a polyvinylidene difluoride membrane (Perkin Elmer, Rodgau, Germany). Blots were blocked with 1% BSA in Tris-buffered saline containing 0.05% tween for 1 h, washed, and incubated with primary antibodies (Table 1) diluted in blocking solution overnight at 4°C. Blots were washed and incubated with HRP-conjugated secondary antibodies (Table 2) for 1 h. HRP activity was detected using ECL Western blotting (Amersham Biosciences) and scanned with DNR Bio-Imaging System and analyzed by using software TINA. The ratios of optical density of the investigated proteins were normalized to *β*-actin.

### 2.6. Statistical Analyses

Statistical analysis was performed with SigmaPlot 12.5 (Systat Software, Erkrath, Germany). Values are given as mean ± SEM. For statistical differences between the two genotypes we used *t*-test or Mann-Whitney rank sum test, depending on the distribution of the data.

## 3. Results

The Purkinje cell degeneration (*pcd*-3j/J) mutant mouse is characterized by the loss of PCs and their axons. The neurons of the DCN and lateral vestibular nucleus (LVN), which are innervated by the cerebellar PCs, are affected as well. In immunohistochemical and biochemical investigations, we observed that the degeneration leads to altered synaptic innervation and ECM conformation in the target areas.

### 3.1. Calbindin D-28k in* pcd* Mice

**Figure 1:**
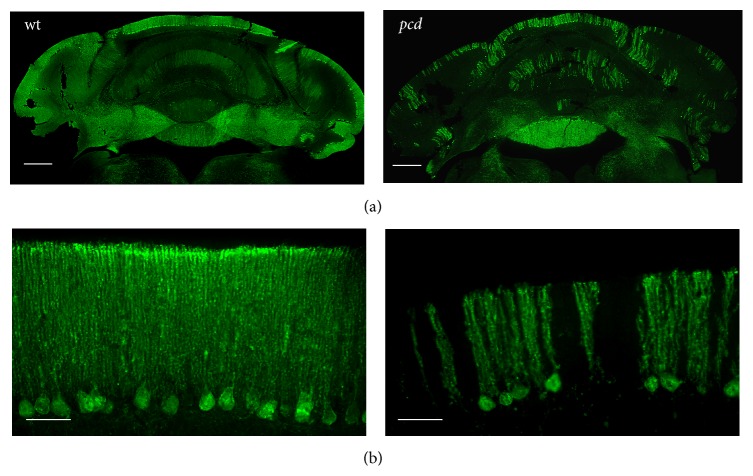
Labelling of calbindin expressing PC neurons in* wt* and* pcd* mice. Purkinje cells and their axons in the cerebellum show a strong immunoreactivity for calbindin. In one month old* wt* anti-calbindin antibodies detect the Purkinje cells and their axon in the cerebellum. The neurons of the cerebellum in one-month-old* pcd* mice reveal less calbindin immunoreactivity. Scale bar: 100 *μ*m (a) and 50 *μ*m (b).

Calbindin* D-28k* is typically used as a marker for the PCs of the cerebellum [20]. The closely spaced somas and axons of the PCs are strongly labeled by antibodies against calbindin. In* pcd* mice this calbindin immunoreaction is significantly reduced. Only very few remaining cells are stained already at one month of age (Figure 1).

### 3.2. Purkinje Cell Degeneration in the Cerebellum Leads to Reduced GABAergic and an Increase of Glutamatergic Synapses in DCN and LVN

Similar to DCN neurons the neurons of the LVN are highly innervated by the GABAergic Purkinje axons. In addition, they receive excitatory input from mossy fibers and climbing fibers [21]. Previous studies demonstrated that pcd is accompanied by a volume reduction and a decrease in cell number in the DCN and LVN with focusing onto inhibitory neurons [22, 73–75]. Thus, we studied if the degeneration of PCs in the cerebellar cortex modifies the terminating synapses of the afferent fibers at the large excitatory PN-bearing projection neurons in the DCN and LVN. Therefore, we investigated the GABAergic terminals by anti-GAD65/67 antibody labeling and the majority of glutamatergic terminals labeled by a mixture of anti-vGlut1 and 2 antibodies at the PN-ensheathed neurons. The different nuclei (DCN, LVN) show similar distribution of these markers. Hence, the data of the nuclei were pooled.

**Figure 2:**
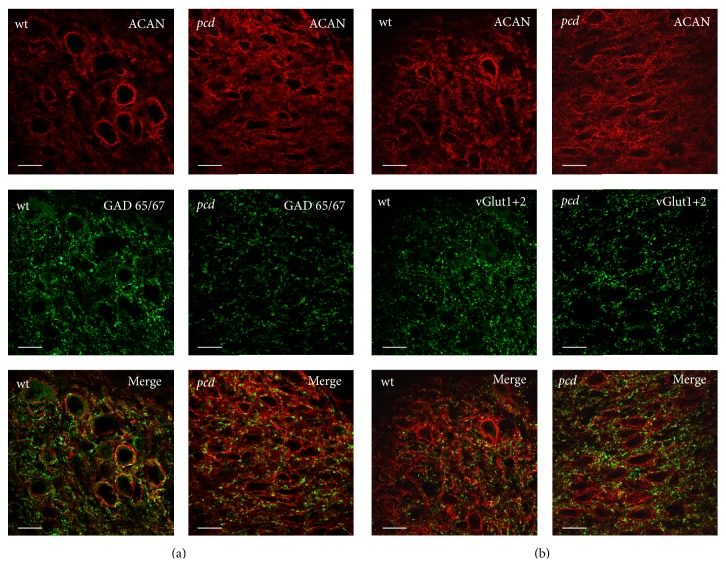
Detection of glutamatergic and GABAergic terminals in DCN. The large DCN neurons are enwrapped by aggrecan-based ECM (red). (a) DCN neurons are innervated by GABAergic boutons, labeled by GAD 65/67. GABAergic terminals seem to be reduced in* pcd*. (b) The glutamatergic boutons at DCN neurons are discovered by moderate vGlut 1 and vGlut 2 staining. The staining in* pcd* appears slightly enhanced. Scale bar: 20 *μ*m.

**Figure 3:**
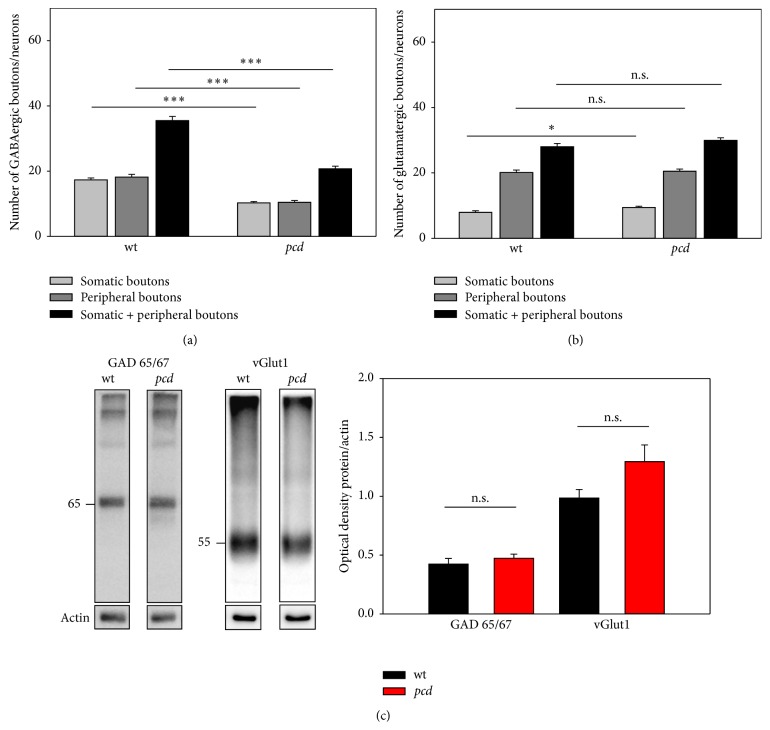
Quantification of GAD65/67 and vGlut in DCN. (a) Quantification shows the distribution of GABAergic terminals in different distances from the PN-bearing neurons. The total number of boutons are reduced in* pcd *compared to* wt*, regardless of the distance. (b) Somatic glutamatergic terminals at DCN neurons appear to be enhanced in* pcd* mice. The peripheral synapses remain unaffected by the insult. (c) Western blot analyses of GAD65/67 and vGlut1 with protein extracts of DCN sections. Typical specific bands are visible in both genotypes. Quantification of these bands reveals slight but no significant differences between* wt* and* pcd *(GAD65/67 *p* = 0.419; vGlut1 *p* = 0.087). Data are given as mean ± SEM.

We could identify a high density of GABAergic terminals at the cell surface (up to 3 *μ*m distance) as well as in the periphery (≥3 *μ*m; for details see Section 2) of each PN-bearing neuron. The number of GABAergic synapses at large PN-ensheathed neurons of* pcd* mice was significantly lower than in the* wt* mice (Figures 2(a) and 3(a); somatic boutons:* wt*: 17.34 ± 0.58 and* pcd*: 10.27 ± 0.41; Mann-Whitney  *U* *p* < 0.0001; peripheral boutons:* wt*: 18.18 ± 0.83;* pcd*: 10.44 ± 0.55; Mann-Whitney  *U* *p* < 0.0001). There were no differences between the densities of somatic versus peripheral GABAergic boutons in both genotypes. Western blot analyses of whole DCN tissue homogenates with anti-GAD65/67 antibody identified the typical molecular weight bands at 65 and 67 kDa, respectively, though the quantification of the GAD band of* wt* and* pcd *showed no significant differences (Figure 3(c);* wt*: 0.423 ± 0.05 and* pcd*: 0.473 ± 0.03  *t*-test *p* = 0.419). 

Beside the inhibitory innervation, the DCN neurons receive excitatory glutamatergic input from collaterals from the mossy and climbing fibers [21, 76]. Immunolabeling of the glutamatergic synapses at PN-bearing neurons reveals that the majority of glutamatergic boutons is not directly located at the soma of these neurons, but rather in their periphery, about ≥3 *μ*m away from the cell body. By quantification of somatic and peripheral terminals, we could verify the differences between somatic and peripheral density of the glutamatergic synaptic terminals (Figure 3(b)). The density of somatic glutamatergic synapses in DCN and LVN of* pcd* mice is increased, while the number of peripheral terminals is not altered (Figures 2(b) and 3(b); somatic boutons:* wt*: 7.93 ± 0.51 and* pcd*: 9.43 ± 0.38; Mann-Whitney *U* *p* = 0.010; peripheral boutons:* wt*: 20.08 ± 0.77;* pcd*: 20.50 ± 0.67; Mann-Whitney *U* *p* = 0.084). In addition, the quantification of the 55 kDa anti-vGlut1 immunoreactive band in western blot confirmed an increase of vGlut1 in* pcd* mice without reaching significance (Figure 3(c);* wt*: 0.985 ± 0.07 and* pcd*: 1.295 ± 0.14; *t*-test *p* = 0.087).

### 3.3. ECM Composition in DCN of* pcd* Mutant Mice

The axons of the Purkinje cells are the sole output of the cerebellar cortex and innervate the neurons of the DCN. The DCN mainly contain 2 types of neurons: large excitatory and smaller inhibitory neurons [53, 77]. As previously described, the large neurons of the DCN are ensheathed by very prominent PNs [25, 58]. The DCN neurons in* wt* mice express the major ECM components aggrecan, brevican, neurocan, tenascin-R, hyaluronan, and hapln [25].

#### 3.3.1. Brevican

**Figure 4:**
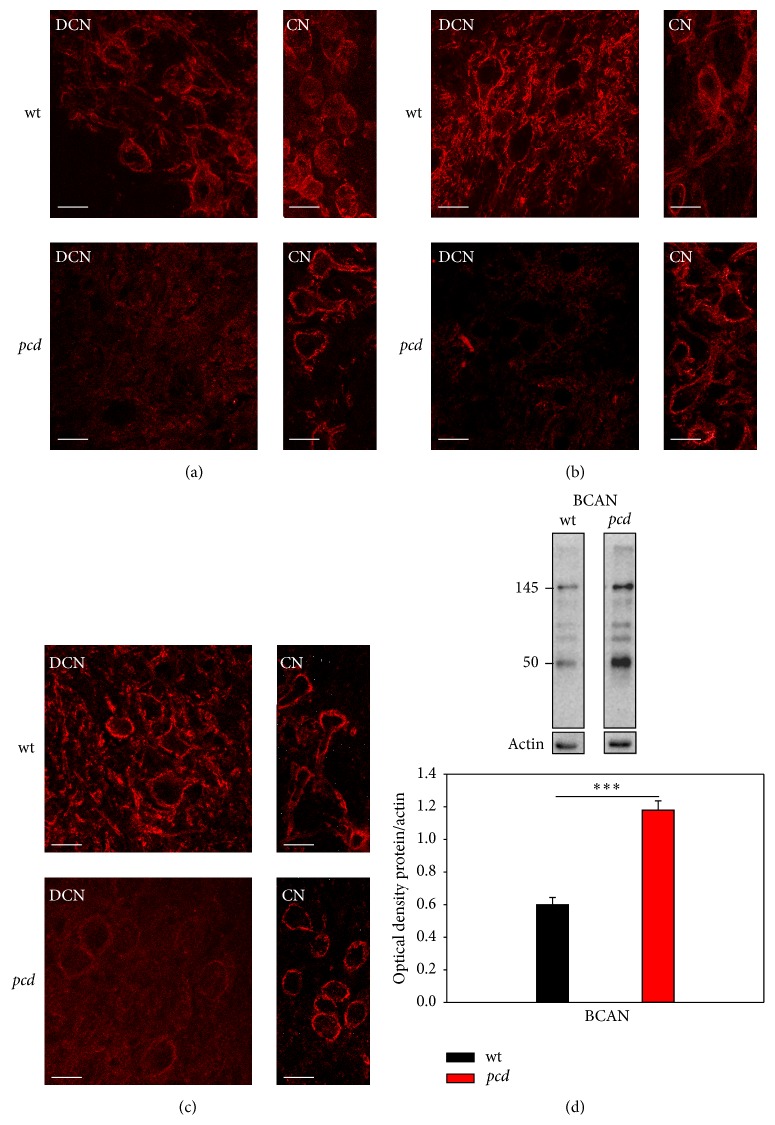
Detection of different brevican fragments. (a) Immunoreaction with pan-specific brevican antibodies (BD Bioscience, FL) clearly surrounds DCN neurons in* wt *but not in* pcd* mice. As internal control nontarget region of PC axons, the cochlear nucleus shows no alterations in immunoreactions with FL. (b) The 50 kDa isoform of brevican seems to be nearly absent around DCN neurons of* pcd* mice, whereas the not affected region (CN) revealed brevican-bearing neurons. (c) The B756 antibodies detect mainly the 80/90 kDa and full length isoforms of brevican. These cleavage products aggregate around the neurons in DCN of* wt* mice and seem to be integrated in PNs. In* pcd*, PNs appear with lower intensity, but with potential higher parenchymatic reaction. PN-detection with all three antibodies in the internal nontarget control region (CN) is unchanged. Scale bar: 20 *μ*m. (d) Biochemical detection of brevican with SDS-PAGE with pan-specific antibodies revealed most known isoforms at 50, 80, 90, and 145 kDa. Quantification of the 50 and 145 kDa brevican isoform showed a significant increased protein expression in* pcd *(*p* < 0.001), respectively, for the different isoforms. Therefore, the diagram is supposed to display the optical density (OD) values of pan-brevican chemiluminescent signal summed values (OD 50 kDa + 145 kDa brevican/actin). Data are given as mean ± SEM.

Recently it was shown that the proteoglycan brevican is enriched at perisynaptic sites and is suggested to be associated with synaptic molecules [18, 78]. Brevican has a metalloproteinase specific cleavage site and can occur as 50 and 80/90 kDa cleavage product and as full length protein of 145 kDa with no chondroitin sulfate (CS) and the CS-bearing variant of over 245 kDa [14, 64]. We investigated the incidence of the cleavage products and the CS-free type of full-length brevican* pcd* mice. For immunocytochemistry, three different antibodies against brevican were used: anti-brevican (BD Bioscience, FL) which detects the full length and the cleavage products, anti-B50 detecting exclusively the 50 kDa cleavage product of brevican and anti-B756, which detects mainly the 90 kDa and the full length isoform. In* wt* DCN immunostaining with all brevican antibodies clearly revealed an immunoreactivity around the large DCN neurons and illustrates the typical brevican-based PN structure surrounding soma and proximal dendrites. In* pcd* mice the DCN neurons show only very weak anti-B50 immunoreaction. Neurons and dendrites are still surrounded by faint immunoreactivity, whereas neurons in nontarget areas of PC axons like the cochlear nucleus (CN) are not affected and show the typical brevican-based PN structure (Figure 4(b)). The FL and B756 antibodies show similar intensities of immunoreactivity in the* pcd* mice. In the DCN not only the neuronal surface is detected, but the whole extracellular space reveals a slight and uniform immunoreactivity. As mentioned above, the neurons of the CN, as an internal reference, show no alterations in the immunoreactivity with FL and B756. The neurons and proximal dendrites still display the typical brevican-based PN structure in* pcd* mice (Figures 4(a) and 4(c)). To further clarify if the altered immunodetection of brevican in* pcd* mice is caused by reduced protein expression we investigated the protein levels of full length and the 50 kDa cleavage product of brevican by western blot analyses. Surprisingly, a significant increased protein level of brevican could be detected in both the full length as well as the 50 kDa cleavage product in the DCN containing tissue of* pcd* mice compared to* wt* mice. The brevican protein amount in the* pcd* mice almost reached a 2-fold increase compared to* wt* mice (Figure 4(d), BCAN:* wt*: 0.599 ± 0.04 and* pcd*: 1.180 ± 0.06; *t*-test *p* < 0.001).

#### 3.3.2. Link Proteins

Link proteins are known to interact with hyaluronan and CSPGs and stabilize this connection. Hapln1 (Crtl-1) and hapln4 are the two link proteins which are associated with PNs and exclusively expressed by PN-bearing neurons [25, 26, 59]. It is supposed that hapln1 is an important component in PN formation. The upregulation of hapln1 expression correlates with PN development and hapln1 deficient mice showed attenuated PNs [32].

**Figure 5:**
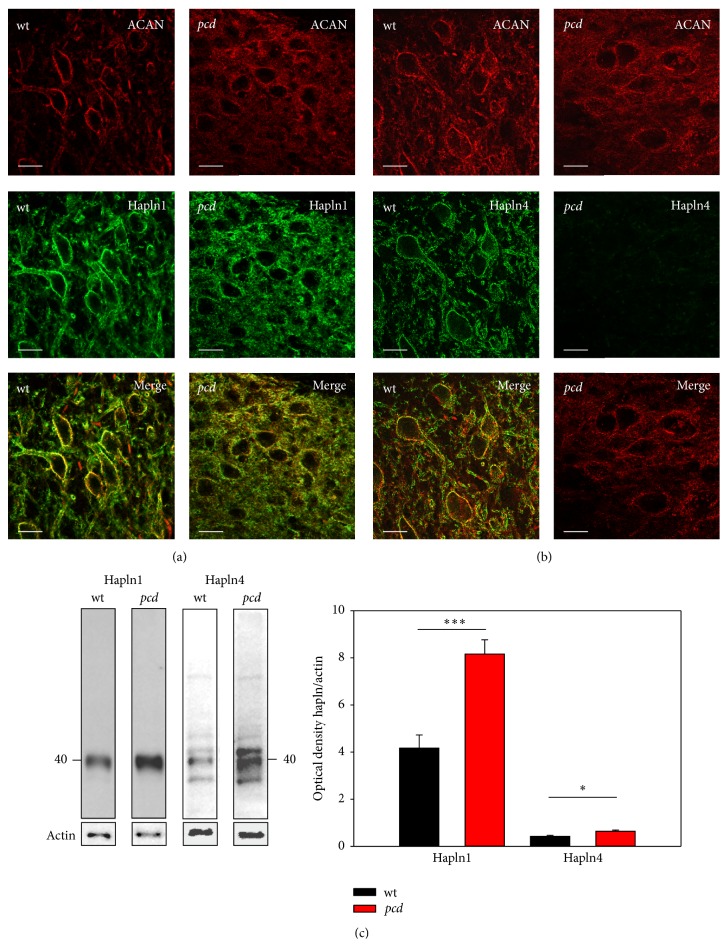
Comparison of link protein expression in DCN. DCN neurons are visualized by aggrecan immunoreaction (red). (a) Hapln1 labeling (green) surrounds the DCN neurons in both genotypes, matching the aggrecan immunoreactivity; additionally in* pcd* hapln1 immunoreaction is distributed throughout the whole parenchyma. (b) Hapln4 (green) encloses the DCN neurons in* wt* mice. In contrast, hapln4 in* pcd* exhibits virtually no immunoreaction. Scale bar: 20 *μ*m. (c) Western blot reveals protein bands at approximately 40 kDa for link proteins. Quantification of the link proteins yielded an elevated protein level of both components in* pcd *(hapln1 *p* < 0.01; hapln4 *p* < 0.05). Data are given as mean ± SEM.

The PN of the large excitatory DCN neurons of* wt* mice is characterized by a strong aggrecan staining and a comparably intensive staining by hapln1. The labeling of both PN-components, aggrecan and hapln1, is for the most part congruent and they seem to be colocalized. In* pcd* mice the detection of aggrecan and hapln1 is less intensive but still clearly present (Figure 5(a)). The immunoreactions of both components appear largely colocalized, although the immunoreactions appear to be redistributed away from the nets into the neuropil and extracellular space, which might be in agreement with immunoblot analyses revealing increased occurrence of hapln1 (Figure 5(c), hapln1:* wt*: 4.164 ± 0.6 and* pcd*: 8.165 ± 0.6; *t*-test *p* = 0.001).

Hapln4 immunodetection also marks PN-bearing neurons in the DCN of* wt* mice. The large DCN neurons are surrounded by delicate hapln4 staining and the labeling is colocalized with the aggrecan immunoreaction. The DCN neurons of* pcd* mice are immunopositive for aggrecan, but virtually no hapln4 immunoreaction is detectable (Figure 5(b)). In contrast, on western blots the hapln4 protein, in DCN enriched homogenate, is slightly significantly elevated (Figure 5(c), hapln4:* wt*: 0.424 ± 0.04 and* pcd*: 0.637 ± 0.05; *t*-test *p* = 0.014).

#### 3.3.3. Hyaluronan and Tenascin-R

**Figure 6:**
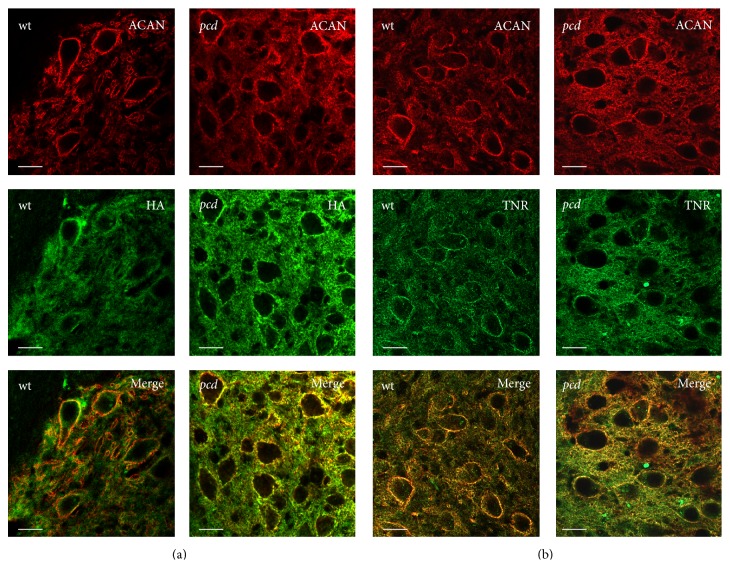
Distribution of hyaluronan and tenascin-R in DCN. The labeling shows important constituents of PNs. The neurons in DCN are surrounded by strong aggrecan immunoreaction (red). (a) Hyaluronan is ubiquitously distributed and concentrated around the neurons. (b) Tenascin-R is equally present in DCN of* wt *and* pcd* mice and mainly encloses the neurons. Scale bar: 20 *μ*m.

Hyaluronan is a very large linear polymer and is supposed to be the backbone of PNs. To visualize hyaluronan in the DCN we used biotinylated hyaluronan binding protein (HABP). Hyaluronan shows a ubiquitous distribution in the DCN of* wt* and* pcd*, with a typical elevated reactivity around PN-bearing neurons, and colocates with aggrecan immunoreaction in PNs (Figure 6(a)). Immunolabeling with tenascin-R reveals similar staining patterns in DCN of both genotypes and show no obvious differences between transgenic and* wt* mice (Figure 6(b)).

### 3.4. Gliosis in DCN after Purkinje Cell Degeneration

**Figure 7:**
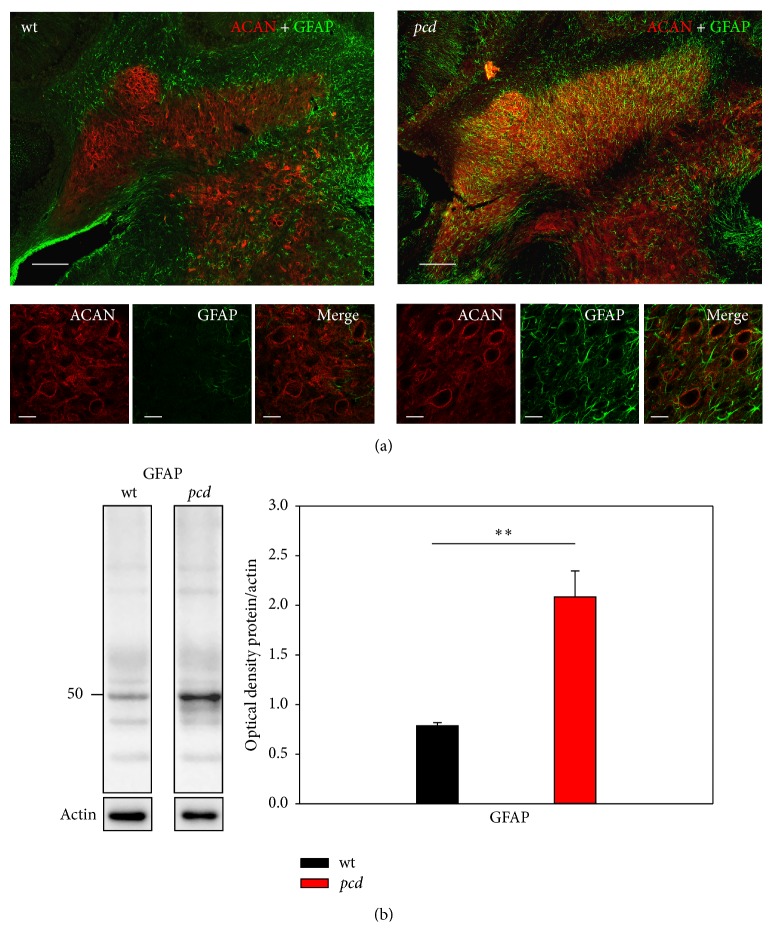
Reactive astrogliosis in the DCN of* pcd* mouse brain. (a) DCN of* wt* mouse brain is characterized by the virtual absence of reactive astrocytes. In* pcd*, the degeneration process is accompanied by a strong astrocytic activation; the DCN seem to be filled with astrocytes. Scale bar: overview 100 *μ*m, detail 20 *μ*m. (b) Western blot analyses confirm the immunocytochemical data. In* pcd* tissue, the GFAP protein level is more than 2-fold increased (*p* < 0.01). Data are given as mean ± SEM.

Lesions and injuries are often followed by gliosis and an increased expression of glial proteins and strong formation of glial structures [79], which replace the degenerated tissue and lost cellular structures. The distribution of astrocytes and their expression level were analyzed with anti-GFAP antibodies, an astrocytic marker. The double immunolabeling with antiaggrecan showed that the* wt* DCN bears only a few GFAP positive astrocytes. In* wt* astrocytes only appear at the edge of the DCN, while the complete DCN of* pcd* mice are marked by a massive glia invasion (Figure 7(a)). These results agreed with the enhanced expression level of GFAP in* pcd* mice and support the assumption that the degeneration of the PCs leads to a strong gliosis in their target area (Figure 7(b), GFAP:* wt*: 0.785 ± 0.03 and* pcd*: 2.086 ± 0.26; Mann-Whitney *U* *p* = 0.008).

## 4. Discussion

### 4.1. Synaptic Input

DCN and LVN neurons are the direct targets of the cerebellar Purkinje cell axons. After their degeneration at an age when nearly all PCs degenerated, the GABAergic terminals in the target regions are significantly reduced [22, 43, 80]. At PN-bearing neurons in DCN and LVN, a subset of large excitatory neurons, the GABAergic terminals are affected as shown by previous investigators [22, 80]. Terminals at the PN-positive neurons are decrease down to 40% independent from terminal localization; somatic terminals as well as peripheral boutons are similarly reduced. In contrast, glutamatergic terminals are increased at PN-bearing neurons after pcd. The degeneration of the PCs and the granule cells in the cerebellar cortex seems to result in a significant reorganization of the synaptic input (reviewed in [75]). Glutamatergic synapses in DCN mainly derive from mossy fibers, which additionally innervate granular cells in cerebellar cortex [81]. Strazielle et al. postulated for another* pcd* model that the loss of Purkinje and granule cells leads to enhanced mossy fiber innervations at DCN neurons [82]. The* pcd-3j/J* model used in this study is also accompanied by an additional decline of cerebellar granule cells. Hence, a similar modification could take place in the DCN of the* pcd* mice and might explain the increased glutamatergic innervations. The excitatory input of the LVN derives mainly from the fastigial nucleus of the DCN [83]; thus the enhanced glutamatergic projection in LVN might be a secondary effect of the lacking inhibition in the DCN neurons. The missing inhibitory innervation and the increased excitatory input might be interpreted as an altered activity of these neurons that could provide an explanation for the ataxic motion. However, in the VN of* pcd* mutants neither the spontaneous activity nor the evoked activation of the neurons are altered ([84, 85] reviewed in [75]). This is in agreement with the observation of axonal sprouting with flat vesicle terminals at the* pcd* DCN neurons [80], which are known to represent inhibitory synapses [86]. The lost GABAergic contacts could be replaced by new non-GABAergic terminals. The massive gain and enhancement of glycinergic boutons observed in DCN and VN could maybe balance the deafferentation of GABAergic axons [74, 80]. Glycine seems to play a predominant role in inhibition and modulates the excitation of DCN neurons; this could temper the symptomatology of the mutants.

### 4.2. Modifications in PN Formation after pcd

The large neurons in DCN are surrounded by PNs composed of hyaluronan, CSPGs, tenascin-R, and link proteins hapln1 and hapln4 [25, 59]. Most of the PN-components are produced by neurons and several are synthetized by glia as well. Decline of Purkinje cells and consequently deafferentation of DCN neurons induce a significant reduction of certain PN-components around the PC-target cells. PNs themselves are still present consisting of the main components hyaluronan, aggrecan, tenascin-R, and hapln1, but brevican and hapln4 are apparently absent. That points to the assumption that brevican and hapln4 are involved in synaptic stabilization and/or maintenance [78, 87]. Brevican is typically enriched at perisynaptic sites and is suggested to accumulate molecules necessary for synaptic formation and preservation [14, 18, 87].

It has been reported that brevican expression is altered after brain injuries [15, 88, 89] and a loss of synapses is associated with a loss of brevican and hapln4 or vice versa [8, 87]. In case of pcd the decay of cells and their axons in cerebellar cortex results in altered expression of brevican and hapln4 in the DCN.

The fact that brevican and hapln4 seem to be no longer an integral part of the PNs in the DCN of the* pcd* mice might confirm the assumption that they might be more sensitive to degeneration than the other PN-components and that both components seem to be strongly dependent on each other for integration into the PNs [90, 91]. In early development, most CSPGs are more soluble and have only a low affinity to bind hyaluronan [92, 93]. Link proteins play an important role in promoting the connection of CSPGs to hyaluronan by inducing conformation changes at the CSPGs allowing a strong interaction with hyaluronan [59, 94]. Hapln4 in the DCN is supposed to derive mainly from the PC axons [25, 59, 87], so in* pcd* mice the supply of the link protein is interrupted and potentially affects the localization of brevican [59].

### 4.3. Enhanced Gliosis Determines Protein Properties

CNS injury or degeneration processes are often combined with cell death leading to secretion of molecules triggering an extensive glial response and activation. The activation of different glial cells mostly follows a specific timeline. The first response to acute injuries is the migration of macrophages and microglia, followed by an activation of oligodendrocytes, and finalized with the proliferation of astrocytes [95]. While in the area of insult (cerebellar cortex) in* pcd* mice an activation of microglia and astroglia was observed [96], this study identified that the DCN as a secondary affected area has rare microglia (IBA1 and S100b, data not shown) but extensive astrogliosis. Reactive astrocytes play an important role in regeneration by occupation of the vacated space, uptake of potentially excitotoxic glutamate, stabilization of extracellular fluid and ion balance, and protection from oxidative stress [97]. Beyond these functions, the expression of CSPGs in the injured brain is strongly upregulated due to astrocytes [98]. In regard to these findings several studies focused on brevican, which is expressed by reactive glia in response to brain injuries [14, 92, 93, 97, 99–107]. In the* pcd* mice the SDS-PAGE also reveals an elevation of brevican in DCN after denervation. It is supposed that the cellular source of brevican can switch after injuries and is predominantly produced by astrocytes [14, 108]. Brevican is sensitive to a number of matrix metalloproteinases (MMPs) and a disintegrin and metalloproteinase with a thrombospondin type 1 motif (ADAMTS) creating cleavage fragments of approximately 50 kDa and 80 kDa [64, 109]. In the adult and healthy brain, most MMPs are downregulated. After injuries the expression and enzymatic activity of MMPs and ADAMTS have been shown to be increased [14, 96, 110–118] caused by activated glial cells [118, 119]. MMP9 expression that is enhanced in the cerebellum of* pcd* mice [96] is linked with the growth-associated protein GAP43 and promotes nerve regeneration and axonal sprouting [4, 120, 121]. The increased expression of the 50 kDa cleavage fragment of brevican discovered in this study implies that there might be an increase of protease activity after degeneration in DCN contributing to extracellular matrix proteolysis, which is what loosens the PNs around the DCN neurons and facilitates new sprouting and synaptogenesis [4, 14, 107, 120].

It is not yet clarified if link proteins, which are mainly expressed by neurons, could also switch to glial expression after injury. In* pcd* mice not only the nna1 gene is disrupted, it is reported that the general gene transcription is downregulated [122, 123]. Furthermore inflammatory events, which are known to be associated with degeneration, cause an increased methylation of the genomic region of hapln4 and potentially decrease the neuronal gene expression [93, 124]. The inhibited neuronal gene expression could promote a potential glial expression of hapln4. Sim et al. suggest that a glial hapln4 expression leads to altered protein properties with a rather soluble nature [93]. However, it is supposed that the glial produced brevican is strongly associated with fibronectin which is highly enriched in cerebellum of* pcd* mice [96] and modulates the cell adhesion and motility [93, 104] which may enable the reinnervation of PN-bearing DCN neurons. We speculate that the high amount of brevican could indeed stimulate the expression of the link protein, but hapln4 has no binding partner anymore and cannot be robustly integrated into PNs.

### 4.4. Technical Consideration

Injuries, diseases, and degeneration-processes often lead to changes of PN-composition. It was reported that remodeling sometimes induces enhanced CSPG expression [88, 108, 125, 126], but it has also been shown that loss of synapses could be associated with reduced CSPG occurrence [4, 8, 87]. Our data display both reduction and enhancement of PN-constituents after degeneration. Deepa et al. [60] showed that detectability is strongly dependent on the solubility of the proteins and the used method. Immunohistochemical methods rather detect proteins in stable complexes and discover soluble fractions less efficiently. In contrast, pretreatment of the tissue for SDS-PAGE with special buffer releases most protein fraction, membrane associated, and soluble fractions [60]. The different techniques could lead to different but not contradictory results.

## 5. Conclusions

Degeneration of cerebellar Purkinje cells affects large PN-bearing DCN neurons. The following events are an interplay of degeneration and regeneration. Not only the Purkinje cell derived GABAergic terminals decrease, but also the PN-components brevican and hapln4 are virtually absent in the stable structure of the PNs of DCN neurons in* pcd* mice. Simultaneously, the hapln4 and brevican protein expression is increased, probably caused by severe local inflammation processes with migrating astrocytes.

On one hand the attenuated PNs imply that brevican and/or hapln4 are more sensitive to degeneration and might play a vital role in synaptic reorganization and the loss or variation of them might enable new sprouting of synapses. On the other hand, glial produced PN-components reveal altered properties and could influence cell adhesion and motility to facilitate axonal path-finding.

## Figures and Tables

**Table 1:** Antibodies and markers.

Marker	Detected Component	Source	Dilution		Reference
			IHC	WB	
PN-constituents					
Human antiaggrecan (HAG, clone 7D4)	N-terminal aggrecan, core protein (AGG)	Serotec	1 : 10		[61]
Antiaggrecan (AB1031)	Amino acids 1177–1326 of mouse aggrecan	Millipore	1 : 200		[63]
Antibrevican (B50)	50 kDa cleavage fragment of brevican, brain enriched hyaluronan-binding protein	Dr. R. Matthews	1 : 2000	1 : 2000	[64]
Antibrevican (756)	Mainly 90 kDa cleavage fragment of brevican, brain enriched hyaluronan-binding protein	Dr. R. Matthews	1 : 1000		
Antibrevican (610894)	80 kDa N-terminal fragment and full length up to 145 kDa	BD Bioscience	1 : 1000	1 : 1250	[65]
Biotinylated hyaluronic Acid Binding Protein (bHABP)	Hyaluronan	Cape Cod	1 : 100		[66]
Antihyaluronan and proteoglycan link protein 1 (HAPLN1/Crtl-1)	NS0-derived rhHAPLN1	R&D Systems	1 : 400	1 : 1000	[67]
Antihyaluronan and proteoglycan link protein 4 (HAPLN4)	NS0-derived recombinant human HAPLN4. Gln30-Val402	R&D Systems	1 : 500	1 : 1000	
anti-tenascin-R (clone 619)	Protein backbone of tenascin-R	R&D Systems	1 : 100		[67]
Glial marker					
Glial fibrillary acid protein (GFAP)	a 50 kDa intracytoplasmic filamentous protein in astrocytes	Dako	1 : 1000	1 : 3000	[68]
Transmitter					
Antivesicular glutamate transporter 1 (vGlut1, #135304)	Glutamate transporter 1 in the membrane of synaptic vesicles	Synaptic Systems	1 : 500	1 : 5000	[69]
Antivesicular glutamate transporter 2 (vGlut2, #135404)	Glutamate transporter 2 in the membrane of synaptic vesicles	Synaptic Systems	1 : 500		[69]
GAD65/67	C-terminal region of human GAD 65 and GAD 67	Sigma	1 : 5000	1 : 15000	[61]
Calcium binding Proteins					
Anticalbindin (AB1778)	Recombinant calbindin	Millipore	1 : 1000		[70]
Cytoskeletal proteins					
Anti-*β*-actin	*β*-cytoplasmic actin N-terminal peptide	Sigma-Aldrich		1 : 10000	[71]

**Table 2:** Used secondary antibodies.

Antibody	Marker	Dilution	Source
Streptavidin	Cy3	1 : 250	Dianova
Donkey-anti-mouse	Cy2, Cy3	1 : 1000	Dianova
Donkey-anti-guinea pig	Cy3	1 : 1000	Dianova
Donkey-anti-rabbit	Cy2, Cy3	1 : 1000	Dianova
Donkey-anti-rabbit	HRP	1 : 10.000	DAKO
Donkey-anti-mouse	HRP	1 : 10.000	DAKO
Rabbit-anti-guinea pig	HRP	1 : 10.000	GE Healthcare
Rabbit-anti-goat	HRP	1 : 10.000	GE Healthcare
